# 初诊IV期肺癌患者中性粒细胞/淋巴细胞比值与营养不良风险的相关性分析

**DOI:** 10.3779/j.issn.1009-3419.2024.106.06

**Published:** 2024-03-20

**Authors:** Ping XIAO, Hai PAN, Qing MA, Liping SONG, Diansheng ZHONG

**Affiliations:** 300052 天津，天津医科大学总医院肿瘤内科; Department of Medical Oncology, Tianjin Medical University General Hospital, Tianjin 300052, China

**Keywords:** 营养, 肺肿瘤, 中性粒细胞与淋巴细胞比值, Malnutrition, Lung neoplasms, Neutrophil-to-lymphocyte ratio

## Abstract

**背景与目的:**

恶性肿瘤患者往往伴随营养不良，与预后密切相关。中性粒细胞与淋巴细胞比值（neutrophil-to-lymphocyte ratio, NLR）作为体内炎症的指标，能够预测多种疾病的营养不良风险，然而其与肺癌患者营养不良的关系尚不明确。本研究旨在分析NLR与初诊IV期肺癌患者营养不良风险的关系，并进一步确定NLR的截断值以更好地预测患者营养不良的风险。

**方法:**

回顾性分析2019年5月至2021年2月天津医科大学总医院肿瘤内科收治的209例初诊IV期肺癌患者，应用营养风险筛查2002（nutritional risk screening 2002, NRS 2002）量表对其营养状况进行评估，同时收录患者人口学信息、病理、卡氏体能状态（Karnofsky performance status, KPS）评分、体重指数、合并疾病及临床生化指标的资料。分析NLR与营养不良风险的关系，并采用受试者工作特征（receiver operating characteristic, ROC）曲线来确定预测营养不良风险的最佳NLR临界值。应用多因素Logistic回归进一步评估NLR与营养不良风险之间的关系。

**结果:**

初诊IV期肺癌患者存在营养不良风险的比率为36.36%（76/209）。对NLR与NRS 2002营养不良风险评分进行分析发现，NLR与营养不良风险评分呈正相关（r=0.765, P<0.001）。通过ROC曲线分析，确定初诊IV期肺癌患者营养不良风险的最佳截断值NLR为3.94[曲线下面积（area under the curve, AUC）=0.747，95%CI: 0.678-0.815，P<0.001]，敏感性为55%，特异性为86%，阳性预测值为68%，阴性预测值为77%。相比于NLR≤3.94组的患者，NLR>3.94组的患者更容易发生营养不良（69.49% vs 23.33%, P<0.001），并且NLR为初诊IV期肺癌患者营养不良风险的独立危险因素。

**结论:**

NLR在初诊IV期肺癌患者中与营养不良风险有关，NLR可作为IV期肺癌患者营养风险筛查的指标之一。

## Abstract (en)

**Background and objective:**

Malnutrition is commonly associated with poor prognosis in patients with malignant tumors. The neutrophil-to-lymphocyte ratio (NLR) is an indicator of inflammation in the body and predicts the risk of malnutrition in a variety of diseases; however, its association with malnutrition in lung cancer patients is unclear. The aim of this study is to clarify the association between NLR and nutritional status in stage IV primary lung cancer and to further determine the optimal NLR cut-off that best predicts the risk of malnutrition.

**Methods:**

A retrospective analysis of 209 patients admitted to the Department of Medical Oncology, Tianjin Medical University General Hospital with a primary diagnosis of stage IV lung cancer from May 2019 to February 2021 was performed, and the nutritional risk screening 2002 (NRS 2002) was used to examine their nutritional status. Patient demographic information, pathology, Karnofsky performance status (KPS) score, body mass index (BMI), comorbidities and clinical biochemical indicators were also included. The correlation between NLR and NRS 2002 was investigated. Receiver operating characteristic (ROC) curve was used to determine the best NLR cut-off predi cting malnutrition risk. Multivariable Logistic regression was used to assess the association between NLR and malnutrition risk.

**Results:**

The rate of patients with stage IV primary lung cancer at nutritional risk was 36.36% (76/209). A significant positive correlation was observed between NLR values and NRS 2002 risk score (r=0.765, P<0.001). The ROC curve analysis indicated that an NLR of 3.94 was the optimal cut-off for predicting malnutrition risk (area under the curve=0.747, 95%CI: 0.678-0.815, P<0.001), which showed a sensitivity of 55%, a specificity of 86%, a positive predictive value of 68%, and a negative predictive value of 77%. Patients in the NLR>3.94 group had a significantly higher risk of malnutrition compared to those in the NLR≤3.94 group (69.49% vs 23.33%, P<0.001). Furthermore, NLR was identified as a risk factor for malnutrition in stage IV primary lung cancer patients.

**Conclusion:**

NLR is associated with the risk of malnutrition in stage IV primary lung cancer, and NLR can be used as one of the indicators for screening nutritional risk in patients with stage IV primary lung cancer

恶性肿瘤患者往往伴随营养不良，而晚期肺癌因肿瘤对食管的压迫影响进食、缺氧影响化学感受器对饥饿信号反应的迟钝导致厌食、肿瘤诱发产生的各种生长因子影响代谢水平的异常，以及抗肿瘤治疗产生的不良反应等原因，患者出现营养不良的风险高达30%以上^[1]^。营养不良可导致患者免疫功能降低，无法耐受肿瘤治疗，增加并发症和病死率的发生，与患者的预后密切相关^[2,3]^，因此对肺癌患者进行营养风险评估十分重要。

中性粒细胞/淋巴细胞比值（neutrophil-to-lymphocyte ratio, NLR）可以全面反映身体的炎症状态。NLR升高表明体内中性粒细胞相对增加或淋巴细胞计数相对减少，破坏体内炎症平衡状态，造成肿瘤细胞发生免疫耐受和免疫逃逸，为肿瘤的侵犯转移提供条件。体内炎性信号长期持续存在，促进蛋白质分解代谢，抑制蛋白质的合成代谢，导致合成和分解代谢失衡，与患者营养不良和代谢紊乱密切相关。多项研究^[4,5]^表明NLR与各种疾病患者的营养状况及预后相关，尤其是消化道肿瘤中NLR可以作为营养风险评估指标^[6]^。因此，结合患者血液学相关指标，筛选存在营养不良风险的患者并给予早期干预对患者的预后具有重要意义。然而在肺癌患者中，目前研究^[7⇓-9]^发现NLR与肺癌的预后及免疫治疗疗效相关，但是其与营养不良风险的具体关系尚不清楚。因此本研究拟在初诊IV期肺癌患者中探讨NLR与营养不良风险的关系，并进一步确定NLR的最佳截断值，以更好地预测营养不良的风险。

## 1 资料与方法

### 1.1 一般资料

回顾性分析2019年5月至2021年2月天津医科大学总医院肿瘤内科收治的初诊未经过任何抗肿瘤治疗的IV期肺癌患者，分期依据国际肺癌研究协会制定的第8版肺癌肿瘤原发灶-淋巴结-转移（tumor-node-metastasis, TNM）分期标准，入院24 h内应用营养风险筛查2002（nutritional risk screening 2002, NRS 2002）量表对患者进行营养风险评估。根据NRS 2002评分，患者营养不良风险分为低（<3分）和高（≥3分）两个等级^[10]^。入院24 h内收录患者基本信息、病理、卡氏体能状态（Karnofsky performance status, KPS）评分、体重指数、合并疾病及临床生化指标的资料，计算NLR、淋巴细胞与单核细胞比值（lymphocyte-to-monocyte ratio, LMR）、血小板与淋巴细胞比值（platelet-to-lymphocyte ratio, PLR）等指标。

### 1.2 研究排除标准

无病理、合并感染、长期口服类固醇激素、自身免疫性疾病、既往基础病控制不佳（如疾病处于急性期、合并急性并发症）、3个月内出现急性心脑血管事件、合并血液系统疾病、昏迷、无法配合测量体重、胸腹水、水肿的患者。

### 1.3 统计分析

使用SPSS 19.0进行统计分析。呈正态分布的连续变量使用独立样本t检验进行分析，不符合正态分布的计量资料使用Mann-Whitney检验；分类变量使用卡方检验或Fisher确切概率法进行分析。变量之间的相关性分析使用斯皮尔曼相关系数进行评估。采用单因素Logistic回归分析确定营养不良高风险的危险因素，进一步通过多因素Logistic回归分析确定营养不良风险的独立危险因素，同时计算比值比（odds ratio, OR）和95%置信区间（confidence interval, CI）。使用受试者工作特征（receiver operating characteristic, ROC）曲线评估NLR的灵敏度和特异性。当尤登指数的曲线下面积（area under the curve, AUC）达到最高值时，即为NLR的最佳临界值。所有P值均为双侧，P<0.05为差异具有统计学意义。

## 2 结果

### 2.1 研究对象的临床特征

共纳入209例初诊未经过任何抗肿瘤治疗的临床分期为IV期的肺癌患者，中位年龄66（45-85）岁，男性占70.33%（147/209），女性占29.67%（62/209）；鳞癌占25.36%（53/209），非鳞非小细胞肺癌（包括腺癌、大细胞癌）占52.63%（110/209），小细胞癌占22.01%（46/209）；NRS 2002评分<3分占63.64%（133/209），NRS 2002评分≥3分占36.36%（76/209），初诊IV期肺癌患者存在营养不良风险的比率为36.36%（76/209）。见表1。

**表1:** 根据NLR分组的初诊IV期肺癌患者的临床特征

Variable	Total (n=209)	NLR≤3.94 (n=150)	NLR>3.94 (n=59)	P
Median age (yr)	66 (53-75)	65 (56-75)	67 (53-72)	0.083
Gender				0.402
Female	62 (29.67%)	47 (31.33%)	15 (25.42%)	
Male	147 (70.33%)	103 (68.67%)	44 (74.58%)	
Pathology				0.670
Nonsquamous NSCLC	110 (52.63%)	79 (52.67%)	31 (52.54%)	
Squamous carcinoma	53 (25.36%)	36 (24.00%)	17 (28.81%)	
Small cell carcinoma	46 (22.01%)	35 (23.33%)	11 (18.65%)	
KPS				0.846
≥80	136 (65.07%)	97 (64.67%)	39 (66.10%)	
<80	73 (34.93%)	53 (35.33%)	20 (33.90%)	
HBP	82 (39.23%)	57 (38.00%)	25 (42.37%)	0.560
T2DM	29 (13.88%)	23 (15.33%)	6 (10.17%)	0.331
NRS 2002 score				<0.001
≥3	76 (36.36%)	35 (23.33%)	41 (69.49%)	
<3	133 (63.64%)	115 (76.67%)	18 (30.51%)	
BMI (kg/m^2^)	23.24 (14.53-35.08)	23.74 (17.57-31.5)	21.97 (14.53-35.08)	<0.001
Albumin (g/L)	35 (22-48)	36 (26-48)	32 (22-41)	<0.001
Globulin (g/L)	32 (17-48)	31 (18-48)	34 (17-48)	0.058
LDH (U/L)	207 (111-1912)	203 (114-873)	216 (111-1912)	0.097
Ferritin (ng/mL)	255 (16->2000)	222 (16->2000）	429 (47.4->2000）	<0.001
Creatinine (μmol/L)	61 (25-257)	61 (30-229)	61 (25-257)	0.797
Hb (g/L)	130 (70-179)	130 (74-179)	130 (70-148)	0.148
Platelet (×10^9^/L)	249 (40-685)	242 (51-541)	266 (40-685)	0.060
Neutrophil (×10^9^/L)	4.39 (0.09-18.34)	4.06 (0.09-8.01)	7.25 (2.32-18.34)	<0.001
Lymphocyte (×10^9^/L)	1.47 (0.39-3.39)	1.60 (0.58-3.39)	1.19 (0.39-2.62)	<0.001
Monocyte (×10^9^/L)	0.58 (0.07-1.75)	0.54 (0.24-1.37)	0.69 (0.07-1.75)	<0.001
NLR	3.06 (0.07-16.12)	2.60 (0.07-3.94)	6.00 (4.07-16.12)	<0.001
PLR	166.47 (48.11-628.44)	146.46 (48.11-425.44)	248.36 (60.00-628.44)	<0.001
LMR	2.50 (0.66-15.14)	3.00 (1.20-9.78)	1.50 (0.66-15.14)	<0.001

Data are expressed as median (interquartile range) or percentage (%) or simple frequency as appropriate. NLR: neutrophil-to-lymphocyte ratio; NSCLC: non-small cell lung cancer; KPS: Karnofsky performance status; HBP: high blood pressure; T2DM: type 2 diabetes; NRS 2002: nutritional risk screening 2002; BMI: body mass index; LDH: lactate dehydrogenase; Hb: hemoglobin; PLR: platelet-to-lymphocyte ratio; LMR: monocyte-to-lymphocyte ratio.

### 2.2 分析结果

对NLR与NRS 2002营养评分进行分析发现，NLR与营养不良风险评分呈正相关（r=0.765, P<0.001）。营养不良风险NLR的最佳截断值为3.94（AUC=0.747, 95%CI: 0.678-0.815, P<0.001），其特异性为86%，敏感性为55%，阳性预测值为68%，阴性预测值为77%（图1）。与NLR≤3.94组比较，NLR>3.94组的患者更容易出现营养不良（69.49% vs 23.33%, P<0.001）（表1）。NLR>3.94组较NLR≤3.94组，体重指数、白蛋白、淋巴细胞、LMR更低，中性粒细胞、单核细胞、NLR、PLR更高（表1）。将患者根据营养不良风险分为低（<3分）和高（≥3分）两个等级，应用单因素Logistic回归进行分析，发现年龄、体重指数、NLR、白蛋白、贫血为营养不良风险的危险因素。将单因素有意义的变量进一步做多因素分析，发现NLR为预测初诊IV期肺癌患者营养不良风险的独立危险因素（OR= 4.985, 95%CI: 2.322-10.704, P<0.001）（表2）。

**图1:**
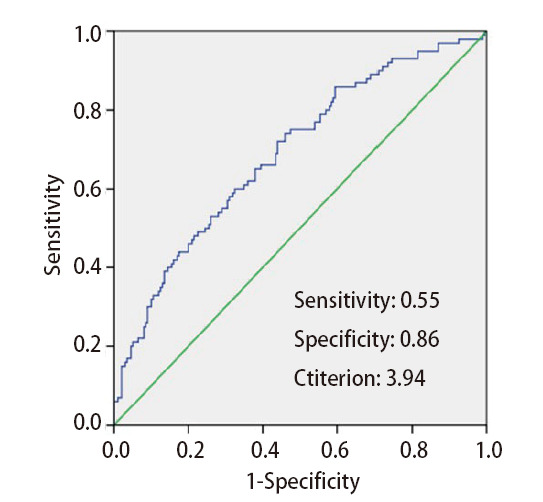
ROC曲线及预测初诊IV期肺癌患者营养不良风险的NLR最佳截断值

**表2:** 根据NRS 2002对营养不良风险进行单因素及多因素Logistic回归分析

Variable		Univariate analysis				Multivariate analysis		
		OR	95%CI	P		OR	95%CI	P
Age (yr)		1.050	1.011-1.091	0.012				
Gender (Male vs Female)		0.947	0.510-1.759	0.864				
BMI (kg/m^2^)		0.766	0.681-0.861	<0.001				
Ferritin		1.001	1.000-1.001	0.050				
NLR (>3.94 vs ≤3.94)		7.484	3.826-14.641	<0.001		4.985	2.322-10.704	<0.001
Albumin(≥30 g/L vs <30 g/L)		0.202	0.098-0.416	<0.001				
Hb (≥120 g/L vs <120 g/L)		0.380	0.206-0.698	<0.001				
Creatinine (μmol/L)		1.001	0.988-1.015	0.857				

OR: odds ratio; CI: confidence interval.

## 3 讨论

本研究表明初诊未经抗肿瘤治疗的IV期肺癌患者营养不良风险比例为36.36%，与既往文献^[2]^报告一致。应用NRS 2002评估营养不良风险在NLR>3.94组和NLR≤3.94组中分别为69.49%和23.33%，并且NLR>3.94为营养不良风险的独立危险因素。

营养不良的评定方法较多，包括身体测量（体重指数、皮褶厚度、体重）、膳食的调查、机体功能的测定（步速、肌力）、机体成分测定（肌肉、脂肪）、实验室检查、复合评定等，不同的工具评估结果并非始终一致。NRS 2002是由欧洲肠外肠内营养学会提出，对于住院患者推荐并倡导使用的营养筛查工具，其简单易行，可以动态、前瞻性地判断患者营养变化，在临床的操作中容易推广^[11]^。目前指南推荐对肺癌患者应用NRS 2002进行营养筛查^[12]^，初步确定存在营养风险的患者，为后续营养干预及治疗提供依据^[13]^。但是对于无法回答问题、意识不清、卧床无法测量体重、存在胸腹水、水肿等影响体重测量的患者，NRS 2002的使用存在限制。

NLR作为一种简单、廉价且有效的方法，结果容易获得，可以弥补NRS 2002的不足。NLR可以反映患者全身炎症，在肝硬化^[4]^、肾功能不全^[5]^、老年住院患者^[14]^、恶性肿瘤^[15]^等多种疾病的临床研究中，发现NLR与患者的营养状况及预后相关。然而不同部位肿瘤、不同疾病状态以及使用不同的营养风险筛查工具，NLR的截断值并不相同。在住院老年患者中，使用微型营养评价法探索NLR与营养不良的关系，发现当NLR>1.81时，预测营养不良风险的敏感性为71.7%，特异性为63.3%（95%CI: 0.562-0.780, P=0.004）^[16]^。在肝硬化患者中，应用专门用于肝病患者的英国皇家自由医院营养优先工具进行营养评分，当NLR>4.2时，其预测营养不良高风险的敏感性为47.2%，特异性为81%（95%CI: 0.55-0.74, P=0.004）^[4]^。而在一项接受手术或放化疗的肿瘤患者的横断面研究^[15]^中使用NRS 2002进行筛查，当NLR>5时，其预测营养不良风险的敏感性为60.9%，特异性为76.4%，不过研究入组的患者以消化道肿瘤和血液系统肿瘤为主。在肺癌患者中，本研究中采用指南推荐的NRS 2002评估肺癌患者的营养不良风险，同样发现NLR升高与患者的营养不良风险相关，并且进一步确定了初诊晚期肺癌患者NLR与营养不良风险的最佳截断值为3.94，其敏感性为55%，特异性为86%。

肿瘤部位、分期、抗肿瘤治疗等情况会影响患者的全身炎症指标与营养状况，进而影响NLR的截断值。因此本研究选择IV期、抗肿瘤治疗前的肺癌患者，尽量避免这些混杂因素的影响。不过本研究为回顾性单中心研究、局限于天津地区，存在人群选择差异，并且样本量较小以及选择偏倚等，这些因素可能会对NLR的截断值有所影响。目前NLR预测营养不良风险的敏感性和特异性有待提高，单个指标的判断能力有限，未来我们需要进一步进行多中心、前瞻性和系统的临床研究，并且可以考虑将全身炎症指标与营养状况评估指标联合，以提高对患者评估的敏感性及特异性。

NLR检测可以发现肺癌中存在营养风险的患者，尽早对其进行营养风险筛查及评估，采取积极的措施提高患者营养及免疫状态，进而改善患者的生存和预后。
